# H-Ferritin-Regulated MicroRNAs Modulate Gene Expression in K562 Cells

**DOI:** 10.1371/journal.pone.0122105

**Published:** 2015-03-27

**Authors:** Flavia Biamonte, Fabiana Zolea, Andrea Bisognin, Maddalena Di Sanzo, Claudia Saccoman, Domenica Scumaci, Ilenia Aversa, Mariafranca Panebianco, Maria Concetta Faniello, Stefania Bortoluzzi, Giovanni Cuda, Francesco Costanzo

**Affiliations:** 1 Department of Experimental and Clinical Medicine, Magna Græcia University of Catanzaro, Salvatore Venuta Campus, Viale Europa, 88100, Catanzaro, Italy; 2 Department of Biology, University of Padua, Via G. Colombo 3, 35131, Padua, Italy; Lady Davis Institute for Medical Research/McGill University, CANADA

## Abstract

In a previous study, we showed that the silencing of the heavy subunit (FHC) offerritin, the central iron storage molecule in the cell, is accompanied by a modification in global gene expression. In this work, we explored whether different FHC amounts might modulate miRNA expression levels in K562 cells and studied the impact of miRNAs in gene expression profile modifications. To this aim, we performed a miRNA-mRNA integrative analysis in K562 silenced for FHC (K562^shFHC^) comparing it with K562 transduced with scrambled RNA (K562^shRNA^). Four miRNAs, namely hsa-let-7g, hsa-let-7f, hsa-let-7i and hsa-miR-125b, were significantly up-regulated in silenced cells. The remarkable down-regulation of these miRNAs, following FHC expression rescue, supports a specific relation between FHC silencing and miRNA-modulation. The integration of target predictions with miRNA and gene expression profiles led to the identification of a regulatory network which includes the miRNAs up-regulated by FHC silencing, as well as91 down-regulated putative target genes. These genes were further classified in 9 networks; the highest scoring network, “Cell Death and Survival, Hematological System Development and Function, Hematopoiesis”, is composed by 18 focus molecules including *RAF1* and ERK1/2. We confirmed that, following FHC silencing, ERK1/2 phosphorylation is severely impaired and that RAF1 mRNA is significantly down-regulated. Taken all together, our data indicate that, in our experimental model, FHC silencing may affect RAF1/pERK1/2 levels through the modulation of a specific set of miRNAs and add new insights in to the relationship among iron homeostasis and miRNAs.

## Introduction

A tight regulation of iron homeostasis is essential for life in eukaryotic cells. The availability of iron is required for critical pathways such as ATP generation and DNA synthesis. Deregulated iron levels contribute indeed to the generation of free radicals that, in turn, damage cellular proteins and nucleic acids [1]. Ferritin, a 24-mer protein, is devoted to keep intracellular iron in a bio-available and non-toxic form [2], thus playing a central role in intracellular iron equilibrium.

The nano-cage of the ferritin molecule is composed by a well-defined array of heavy-type (FHC) and light-type (FLC) subunits, coded by two different genes [3] that, share both extensive aminoacid sequence (55%) and structural similarity. The two subunits perform different functions in iron metabolism: FHC is involved in rapid iron uptake and release and it hasferroxidase activity, while FLC, devoid of enzymatic activity, essentially contributes to long-term iron storage [4]. Recently, several lines of evidence have demonstrated that FHC is a multi-functional protein, that might play a central role in proliferation [5], angiogenesis [6], chemokine signalling [7] and neoplastic transformation [8]. *FHC* expression is modulated, at transcriptional level, by proteins involved in tumorigenesis; among them, E1A [9], p53 [10], and c-Myc [11] act as repressors, while c-Jun is an inducer [12]. FHC itself binds to p53and is able to activate *p53* transcription under oxidative stress conditions [13]. Moreover, *FHC* transcription is activated by TNFα and interleukin 1α (IL-1α) [14], suggesting that pathways related to inflammation and stress can impact on ferritin regulation. The ferritin H subunit also physically interacts with, and regulates the activity of the chemokine receptor CXCR4 [7], highly expressed in a variety of human malignancies. *FHC* down-regulation by shRNA interference strongly modifies, *in vivo* and *in vitro*, the proliferation of human melanoma cells [15].

This scenario is even more complicated when considering the relationship between ferritin and cellular proliferation. *FHC* up-regulation has been associated with induction of differentiation and growth arrest in hematopoietic systems [16], differentiation of theCaco-2 enterocytic cell line [17] and switch from pre-adipocytes to adipocytes [18].

The last decade has witnessed a tremendous increase of knowledge on the role of microRNAs (miRNAs) in regulating gene expression in normal and pathological conditions. These non-coding RNAs, with an average length of 19–25 nucleotides, are able to modulate the expression of thousands of genes by inhibiting translation or inducing degradation of transcripts. Moreover, one target transcript can be controlled by more than one miRNA [19]. It has been suggested that miRNAs might regulate more than 60% of the protein coding genes [20]. Key functional roles for miRNAs have been demonstrated in development, organogenesis and cell differentiation [21]. In hematopoietic stem and progenitor cells miR-221, miR-222, miR-223 and miR-150 act as master regulators, contributing to the hematopoietic development and the lineage specification [22, 23].

The role of miRNAs in cancer has been deeply investigated. Specific patterns of miRNA expression (*miRNome*) and variations have been established in different tumour stages and subtypes. miRNAs can play oncogenic (oncomiRNAs) and/or tumor suppressive role in almost all the aspects of cancer biology [24].Moreover, a specific miRNA can play opposite roles in different contexts: for example, miR-29 acts as a tumor-suppressor in lung cancer, while it plays oncogenic functions in breast cancer [25]. Like virtually all other cellular processes, also iron homeostasis is regulated by specific miRNAs. miR-210 acts on the transferrin receptor and is involved in iron acquisition. Iron storage and utilization are controlled by miR-200b, targetingFHC, while iron release is regulated by miR-485-3p, through its action on ferroportin (Fpn) [26].

We have recently found that, in a metastatic melanoma cell line [15] and in the K562 erythroleukemia cell line [27], the silencing of *FHC* subunit is accompanied by profound modifications of gene expression. The molecular basis of the link among *FHC* levels and gene expression profile in these cells have not been established yet.

In this study, we profiled both mRNA and miRNA expression in K562 cells silenced for the ferritin H subunit and compared these expression profiles with that of control cells. We identified specific miRNAs and genes differentially expressed upon *FHC*-knock down and studied the relations thereof.

## Materials and Methods

### miRNA isolation and quantitative real-time PCR

miRNA-enriched total RNA was extracted from a pool of cultured FHC-silenced K562 (K562^shFHC^) cells and K562 transduced with scrambled RNA (shRNA) using miRCURYRNA Isolation Kit Cell and Plant (EXIQON, Woburn, USA) following the manufacturer’s protocol. The concentration of RNA and the RNA quality (260/280 and 260/230 absorbance ratios) of the samples were measured using Nanodrop (Thermo SCIENTIFIC, Waltham,MA,USA). We designed a double-step analysis for identification and quantification of abnormally expressed miRNAs in K562 shFHC compared to K562 shRNA cells. The first was a “panel” procedure that simultaneously evaluated expression level of different maturemiRNAs by quantitative real-time PCR (qRT-PCR); the second was performed on individual miRNAs, which eventually resulted differentially expressed in panel experiments. For panel analysis, we used Cancer Focus microRNA PCR Panel that assesses the expression levels of 84 onco-miRNAs.

Each sample was assayed in triplicate, and the experimental data were normalized to the expression levels of the housekeeping small nuclear RNA,U6.

### Identification of differentially expressed miRNAs

The fold change of miRNAs expression among the tested samples was calculated using 2^−ΔΔCt^formula. Differences among the two sets of samples were analyzed by the Student*t*-test. Those differences with a p<0.05 were considered statistically significant.

From this first analysis we decided to focus on those miRNAs that were found to be up-regulated in K562 shFHC compared to K562 shRNA cells. cDNA synthesis, was performed using TaqManMicroRNA Reverse Transcription Kit (Life Technologies, Carlsbad,CA,USA) containing microRNA-specific RT primers and TaqmanmiRNA assay. To measure miRNAs expression levels, 1.33 μL of each cDNA was added to the specific TaqMan microRNA Assay (20X) and TaqMan 2X Universal PCR Master MiX(Life Technologies, Carlsbad,CA,USA). The amplification conditions for miRNAqRT-PCR were the following: 10 min at 95°C, 40 cycles at 95°C for 15s, and 60°C for 60s. The experiments were performed in duplicate and the analysis was performed using the 2^−ΔΔCt^formula.

### Transfection of K562 cells

K562 cells were transfected using electroporation. In particular, over-expression of *FHC* was performed using the expression vector containing the full length of human FHC cDNA (pc3/FHC); transient silencing of K562 cells was obtained using a homemade FHC siRNA, kindly provided by Prof. Sonia Levi from the Vita-Salute San Raffaele University Milano, Italy. For rescue of *FHC* expression, approximately 6×10^6^K562-silenced cells (K562^shFHC^) were resuspended in 600μL of Opti-MEM (Gibco BRL). Subsequently,3×10^6^of cell suspension was mixed with 30 μg of pcDNA3/FHC (K562^shFHC/pc3FHC^). The remaining 3×10^6^of cell suspension was mixed with the *control*plasmid,*pcDNA3*(K562^shFHC/pcDNA*3*.*1*^).For transient silencing of *FHC*, 6x10^6^ K562 cells were resuspended in 600μL of Opti-MEM and then, half was mixed with 15μg of a GFP-positive control siRNA (K562 Ctrl siRNA) and half with 15μg of FHC siRNA (K562 FHC siRNA). After 15 minutes of incubation at room temperature, each samplewaselectroporated in a sterile electroporation cuvette (Bio-Rad Gene Pulser cuvette, 0.4 cm) using Gene PulserXcell Electroporation System (Bio-Rad). Electroporation was performed at 285V and 975μFa. After electroporation, cell suspensions were centrifugated at maximum speed and the pellets were left at room temperature for 20 minutes. Then, fresh complete medium was added to the pellets and cells were further incubated at 37°C in a humidified atmosphere supplemented with 5% CO2. Transfection efficiency was measured after 72h using real-time PCR.

### Identification of differentially expressed genes

Genes modulated after FHC silencing have been identified using Limma package [28]. Differential expression analysis was obtained by a t-statistic, which is computed for each gene and for each contrast, with standard errors moderated across genes, exploiting the Empirical Bayes shrinkage method to stabilize the variance estimate.

Only genes with absolute log(FC) of at least 1 and a FDR q-value lower than 0.1 have been considered differentially expressed.

### Identification of anticorrelated predicted targets of miRNAs

We identified the predicted regulatory relations significantly supported by expression data, integrating target predictions with miRNA and gene expression profiles in silenced and un-silenced cells. Only differentially expressed miRNAs were considered. Target predictions were computed with TargetScan.

Pairwise Spearman correlations between miRNA and predicted target gene expression profiles were calculated. The supported relationships associated to statistically significant correlations (r< = -0.81 and p-value < = 0.05) were selected.

### Pathways visualization

Network visualization and annotation have been performed using Cytoscape[29].

### Functional analysis of target genes

In order to infer the potential functions of the differentially expressed miRNAs, we performed the functional analysis of their target genes using Ingenuity Pathway Analysis (IPA) database.IPA maps each gene within a molecular network and defines it as “focus molecule”. Ingenuity Pathway Analysis (IPA) software program was used as described elsewhere [30]. Following IPA analysis, Panther (Protein ANalysisTHrough Evolutionary Relationships) was used to also classify genes in specific signalling and metabolic pathways (http://www.pantherdb.org/).

### RNA extraction and quantitative real-time PCR for *FHC* and *c-Myc*and *RAF1*detection

Total RNA was extracted from two distinct batches of K562 shRNA and K562 shFHC cells using the Trizol method (Life Technologies, Carlsbad,CA,USA). Real-time PCR was performed using 10X SYBR Green PCR Master mix (Life Technologies, Carlsbad,CA,USA), 400 nM of each primer pair, 20ng of cDNA (total RNA equivalent) and nuclease-free water. The thermal profile consisted of 1step at 95°C for 10min followed by 45cycles at 95°C for 30s, 60°C for 60s. Human glyceraldehyde 3-phosphate dehydrogenase (GAPDH) was used as housekeeping. Each reaction was performed in duplicate. The primer sequences for *FHC* and *GAPDH* have been already published (27). The primer sequences for *RAF1* and *c-MYC* were as follow:

RAF1 FW: TGCTGCGTCTTTGATTGGAG

RAF1 REV: TGGTGCTACAGTGCTCATGA

c-MYC FW: CCTCGGATTCTCTGCTCTCC

c-MYC REV: TGTGAGGAGGTTTGCTGTGG

### Protein Extraction and Western Blotting Analysis

K562 shRNA and K562 shFHC cells were lysed in the following buffer [20mMHepes pH7.9, 420mMNaCl, 1% Triton X-100, 1mM EDTA, 25% glycerol, 1mM PMSF, 1mM Na_3_VO_4_, 1mM DTT, 1μg/ml aprotinin, 1μg/ml leupeptin] for 30min on ice. After removal of the cell debris by centrifugation (12,000×*g*, 30min), the concentration of proteins in the supernatant was measured by the Bio-Rad protein assay according to the manufacturer's instructions (Bio-Rad Laboratories, Hercules,CA,USA) [31]. A total of 50μg protein extract was boiled for 10min in SDS sample buffer, separated by 12% SDS-PAGE and the proteins were transferred to a nitrocellulose membrane by electroblotting. Non-specific reactivity was blocked by incubating the membrane in nonfat dry milk in TPBS [5% (w/v) milk in PBS (pH7.4) and 0.005% Tween 20] for 2h at room temperature. The membrane was incubated with primary mouse anti-Phospho-p44/42 MAPK (Erk1/2) (Thr202/Tyr204) antibody (1:1000; Cell Signaling Technology, Danvers, MA, USA) overnight at 4°C. Being washed in TPBS, the membranes were subsequently incubated with anti-mouse secondary antibody (1:3000Cell Signaling Technology, Danvers, MA, USA) for 2 hours. The membrane was developed by ECL-Western blot detection reagents according to the manufacturer's instructions (Santa Cruz Biotechnology, Texas, USA). γ-Tubulin was used as a loading control.

### Assessment of cell proliferation

3-[4,5-Dimethylthiaoly]-2,5-diphenyltetrazolium bromide (MTT) assay was performed to detect proliferation of K562 shRNA and K562 shFHC cells. The experiments were performed on starved cells that were obtained culturing proliferating cells with RPMI 1640 without FBS for 24h. A total of 4.5X10^4^ cells/well were seeded into 96-well plate and let to grow for 72h in RPMI medium. There were octuplicates for each cell type. Fresh MTT (Sigma Aldrich, Saint Louis, MO, USA), re-suspended in PBS was added to each well. After 2h incubation, culture medium was discarded and replaced with 200μL of DMSO. Optical density was measured at 570 nm in a spectrophotometer. Each experiment was performed in triplicate.

## Results

### miRNA and transcriptome analysis in K562 cells

Our main goal in the past years has been the identification and classification of genes whose expression is directly or indirectly modulated by FHC in different human cell lines. In the present study, we evaluatedif the silencing of FHC may also alter oncomiRNAs expression in the K562 erythroleukemic cell line with the aim of identifying the potentially regulated genes.To this, we utilized a pool of cell clones, already described, in which FHC expression has been knocked-down with specific shRNA [27]. Using Cancer Focus microRNA PCR Panel, we have identified 59 miRNAs, 12 of which wereup-regulated and 3 down-regulated, with an absolute Log fold-change (LogFC) greater than 1, in K562 cells silenced for H ferritin (shFHC) versus K562 cells transduced with scrambled RNA (shRNA) (S1 Table). The analysis was performed in triplicate from both cell types using quantitative real-time PCR (qRT-PCR). Four miRNAs namely hsa-let-7g-5p, hsa-let-7f-5p, hsa-let-7i-5p and hsa-miR-125b-5p resulted significantly up-regulated, with LogFC variation of at least five and a t-test p-value <0.05, after FHC knock-down (Table 1).

**Table 1:** Four miRNAsare significantly up-regulated after H ferritin silencing.

microRNA	LogFC (shFHCvsshRNA)	p-value
hsa-let-7g-5p	7.38	0.0133
hsa-let-7f-5p	5.47	0.0218
hsa-let-7i-5p	4.95	0.0340
hsa-miR-125b-5p	5.82	0.0470

**Fig 1:**
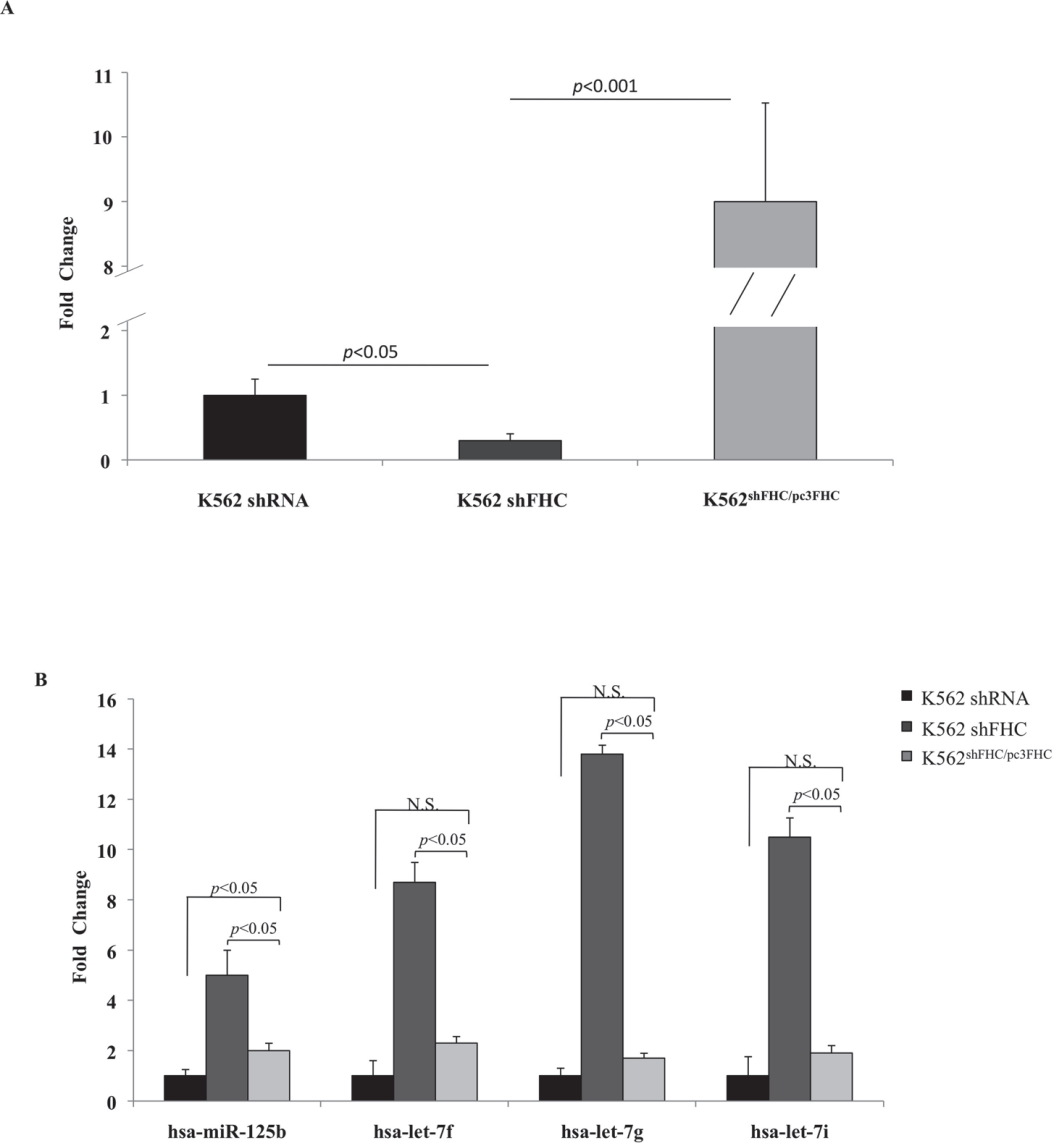
Four miRNAs are significantly modulated by FHC amounts. A) Real-time PCR analysis of FHC mRNA performed on total RNA from K562 shRNA, K562 shFHC and K562^shFHC/pc3FHC^. Results are representative of two different experiments. B) TaqMan analysis of hsa-miR-125b, hsa-let-7f, hsa-let-7g, hsa-let-7i in K562 shRNA, K562 shFHC and K562^shFHC/pc3FHC^. Results are representative of two different experiments. **N.S.: Not Significant**

**Fig 2:**
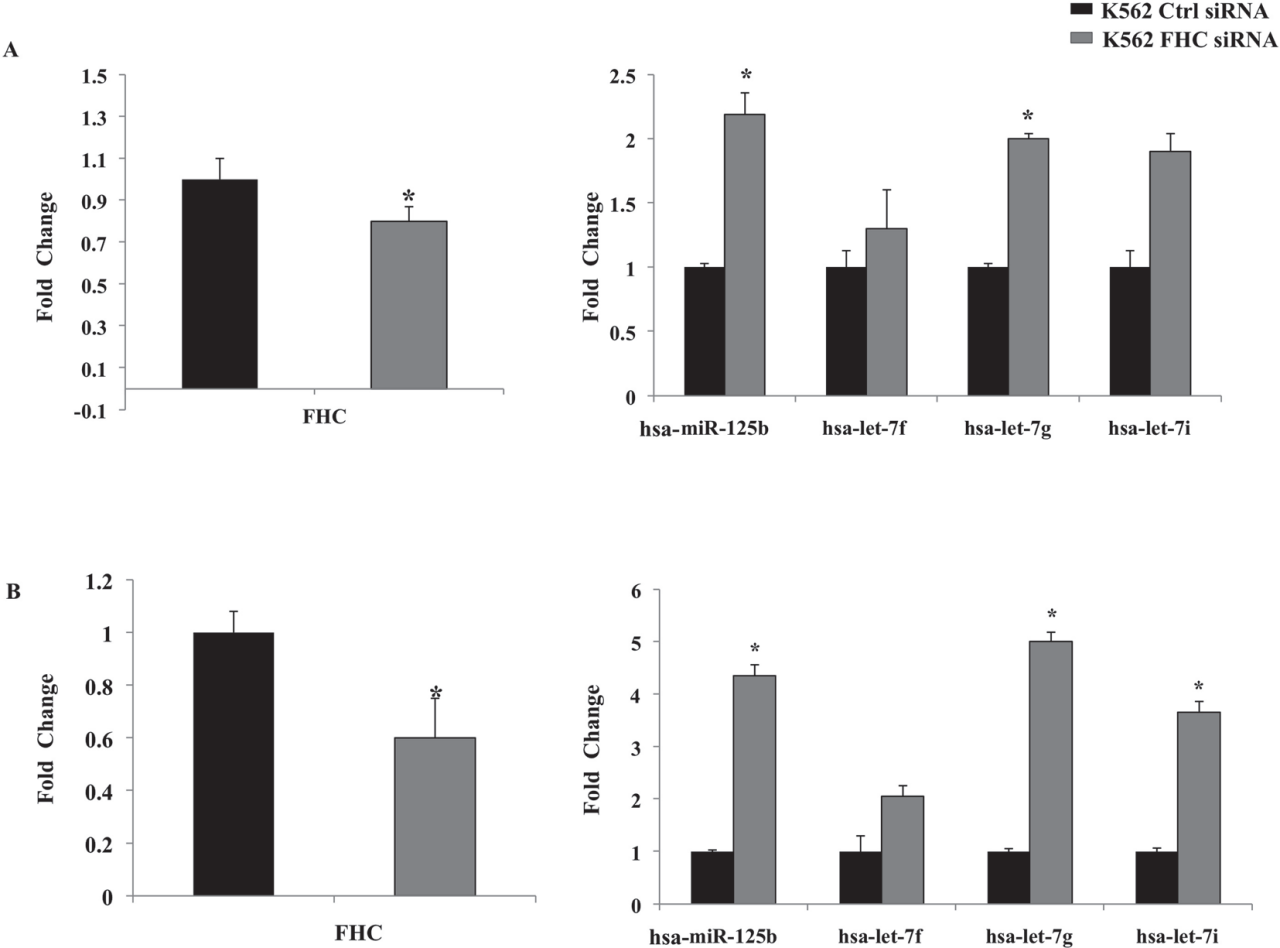
Transient silencing of FHC induce up-regulation of hsa-miR-125b, hsa-let-7f, hsa-let-7g, hsa-let-7i. A transient silencing of FHC of about A) 20% and B) 40% is accompanied by the up-regulation of hsa-miR-125b, hsa-let-7f, hsa-let-7g, hsa-let-7i. Results are representative of two different experiments performed by TaqMan analysis. *p value<0.05

The expression of these four miRNAs was assessed by TaqMan assay in an independent set of RNA obtained from silenced cells and from K562 cells in which the expression of FHC has been restored. The results of a duplicate set of experiments are shown in Panels A and B of Fig. 1. Panel A shows the extent of FHC-silencing and reconstitution. In Panel B are reported the expression levels of the four miRNAs in the silenced and reconstituted K562 cells. It appears that the four miRNAs are indeed up-regulated in the cells in which the FHC subunit was present at a significantly lower level (FC = 0.3) compared to shRNA cells (p-value<0.05) and down-regulated in the cells where FHC expression levels were rescued. According with the microRNA PCR panel, the greatest increase was observed for hsa-let-7g, whose expression is about 14-fold higher in the silenced cells compared to the control. We also transiently trasfected K562 cells with a homemade FHC siRNA kindly provided by Professor S. Levi, and compared the expression levels of the four miRNAs with those of control cells. In two independent experiments, a different silencing efficiency, in the order of about 20 and 40%, was obtained. Panels A and B of Fig. 2 show that, in both samples, *FHC* silencing is accompanied by an up-regulation of hsa-let-7g-5p, hsa-let-7f-5p, hsa-let-7i-5p and hsa-miR-125b-5p.

The gene expression profile of FHC-silenced versus un-silenced K562 cells has been already determined in a previous work [27]. Here, in order to integrate miRNA and mRNA transcriptome findings, we have re-analyzed the raw microarray data. The Limma differential expression analysis identified 219 transcripts with a significantly altered expression in the FHC-silenced cells, including 64 up- and 53 down-regulated genes with an absolute LogFC greater than 2. The full cast of the FHC-dependent mRNAs is reported in S2 Table.

### miRNA-mRNA regulatory network

**Fig 3:**
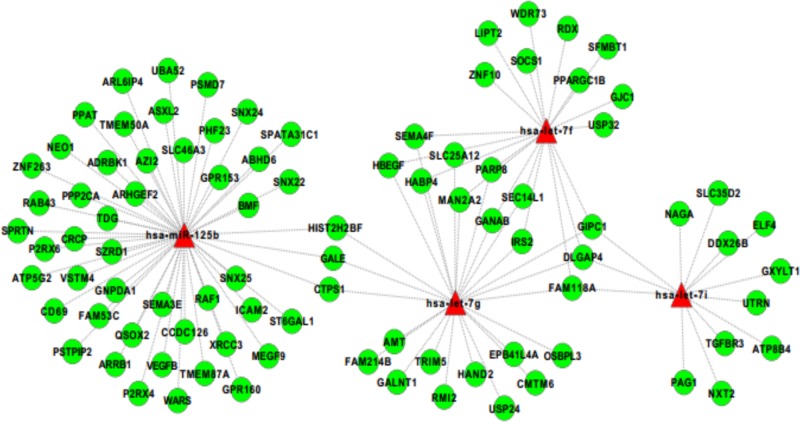
miRNA-mRNA interaction networks. miRNA-mRNA interaction networks built by Cytoscape. We identified a total of 108 miRNA-mRNA significantly negatively correlated interaction. The four up-regulated miRNAs are colored in red and the 91 down-regulated target mRNAs are in green. let-7i is correlated with 13 transcripts; let-7f and let-7g with 20 and 25 transcripts, respectively; miR-125b negatively correlates with 50 transcripts. The majority of genes are supported targets of only one specific miRNA, whereas 15 genes are putatively regulated by two or more distinct up-regulated miRNAs.

Next, for differentially expressed miRNAs, we integrated target predictions with miRNA and gene expression profile, to identify the regulatory relationships significantly supported by expression data. Combining TargetScanpredictions of miRNA-target interactions with a correlation-based analysis of miRNA and transcript expression profiles (see Materials and Methods), we obtained 108 interactions supported by expression data, involving hsa-let-7g-5p, hsa-let-7f-5p, hsa-let-7i-5p and hsa-miR-125b-5p and 91 down-regulated genes. In particular, the expression of hsa-let-7i resulted to be negatively correlated with that of 13 transcripts; hsa-let-7f and hsa-let-7g with that of 20 transcripts and 25 transcripts, respectively; finally, hsa-miR-125b negatively correlated with 50 transcripts. As shown in the reconstructed regulatory network (Fig. 3), the majority of these genes were supported targets of only one miRNA, whereas 15 genes were putatively regulated by two or more up-regulated miRNAs.

The list of the 91 down-regulated transcripts with their cognate miRNAs is reported in S3 Table.

### miRNAs modulated by FHC silencing impact on specific pathways

**Fig 4:**
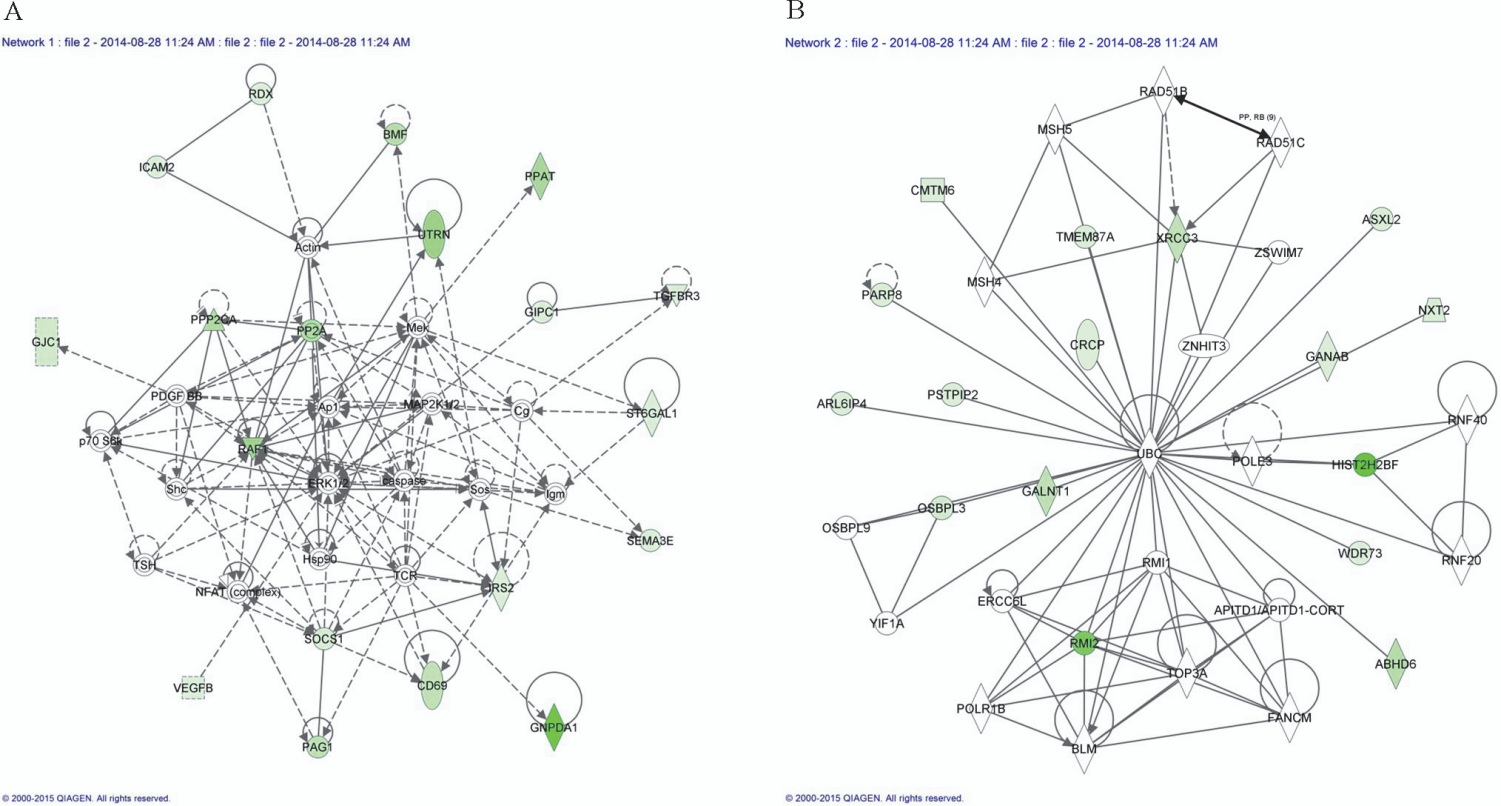
The two highest scoring networks identified by IPA, that correlate genes target of the miRNAs differentially expressed after FHC silencing. Ingenuity Pathway Analysis was used to investigate the networks potentially affected by the down-regulated genes. (A) Cell Death and Survival, Hematological System Development and Function, Hematopoiesis” is the highest scoring network with a significance score of 37 and 18 focus molecules (B) DNA Replication, Recombination and Repair, Cell Cycle, Cancer” has a significance score of 32 and 16 focus molecules. The target down-regulated genes are shaded in green. Intensity of shading correlates with the degree of down-regulation. A solid line represents a direct interaction between two genes, while a dotted line indicates an indirect interaction.

The 91 down-regulated supported target genes of hsa-let-7g-5p, hsa-let-7f-5p, hsa-let-7i-5p and hsa-miR-125b-5p were studied with two knowledge-based approaches to better characterize the networks potentially modulated by *FHC* silencing. Ingenuity Pathway Analysis tool (IPA) highlighted the 9 networks reported in Table 2; of them, the highest scoring is “Cell Death and Survival, Hematological System Development and Function, Hematopoiesis” with a significance score of 37 and 18 focus molecules (Panel A of Fig. 4), followed by “DNA Replication, Recombination and Repair, Cell Cycle, Cancer” with a significance score of 32 and 16 focus molecules (Panel B of Fig. 4). The significance scores of these networks (estimating the probability that a collection of genes equal to or greater than the number in a network can be achieved by chance alone) are very high, since a score of 3 indicates a 1/1000 chance that the focus genes are in a specific network due to random chance.

**Table 2:** Top 9 molecular networks predicted by IPA, by analysis of genes with expression profiles significantly negatively correlated with that of miRNAs differentially expressed after FHC silencing.

Molecules in Network	Score	Focus Molecules	Top Diseases and Functions
BMF, CD69, GIPC1, GJC1, GNPDA1, ICAM2, IRS2, PAG1, PPAT, PPP2CA, RAF1, RDX, SEMA3E, SOCS1, ST6GAL1, TGFBR3, UTRN, VEGFB	37	18	Cell Death and Survival, Hematological System Development and Function
ABHD6, ARL6IP4, ASXL2, CMTM6, CRCP, GALNT1, GANAB, HIST2H2BF, NXT2, OSBPL3, PARP8, PSTPIP2, RMI2, TMEM87A, WDR73, XRCC3	32	16	DNA Replication, Recombination and Repair, Cell Cycle, Cancer
DDX26B, GXYLT1, PHF23, QSOX2, RAB43, SLC46A3, SNX22, SNX24, SNX25, SZRD1, TMEM50A, VSTM4, ZNF10	24	13	Cancer, Gastrointestinal disease, Cell death and Survival
ADRBK1, ARHGEF2, ARRB1, ATP5G2, AZI2, HAND2, HBEGF, P2RX4, P2RX6, SFMBT1, SLC25A12, UBA52, USP24	23	13	Cardiovascular system development and function, Developmental disorders, Organ morphology
AMT, DLGAP4, ELF4, EPB41L4A, FAM214B, MEGF9, SEMA4F, SPRTN, USP32, ZNF263	16	10	Cancer, Gastrointestinal disease, Cell to cell signaling and interaction
CCDC126, FAM118A, FAM53C, GALE, GJC1, GPR153, GPR160, MAN2A2, NEO1, PPARGC1B	16	10	Cancer, Cellular Movement, Tissue Morphology
CTPS1, HABP4, NAGA, PSMD7, SEC14L1, TDG, TRIM5, WARS, LIPT2	13	8	Carbohydrate Metabolism, Developmental Disorder, Hereditary Disorder
LIPT2	2	1	Organ, Morphology, Riproductive System Development and Function, Endocrine System Development and Function
ATP8B4	2	1	Cancer, Organismal Injury and Abnormalities, Reproductive System Disease

**Fig 5:**
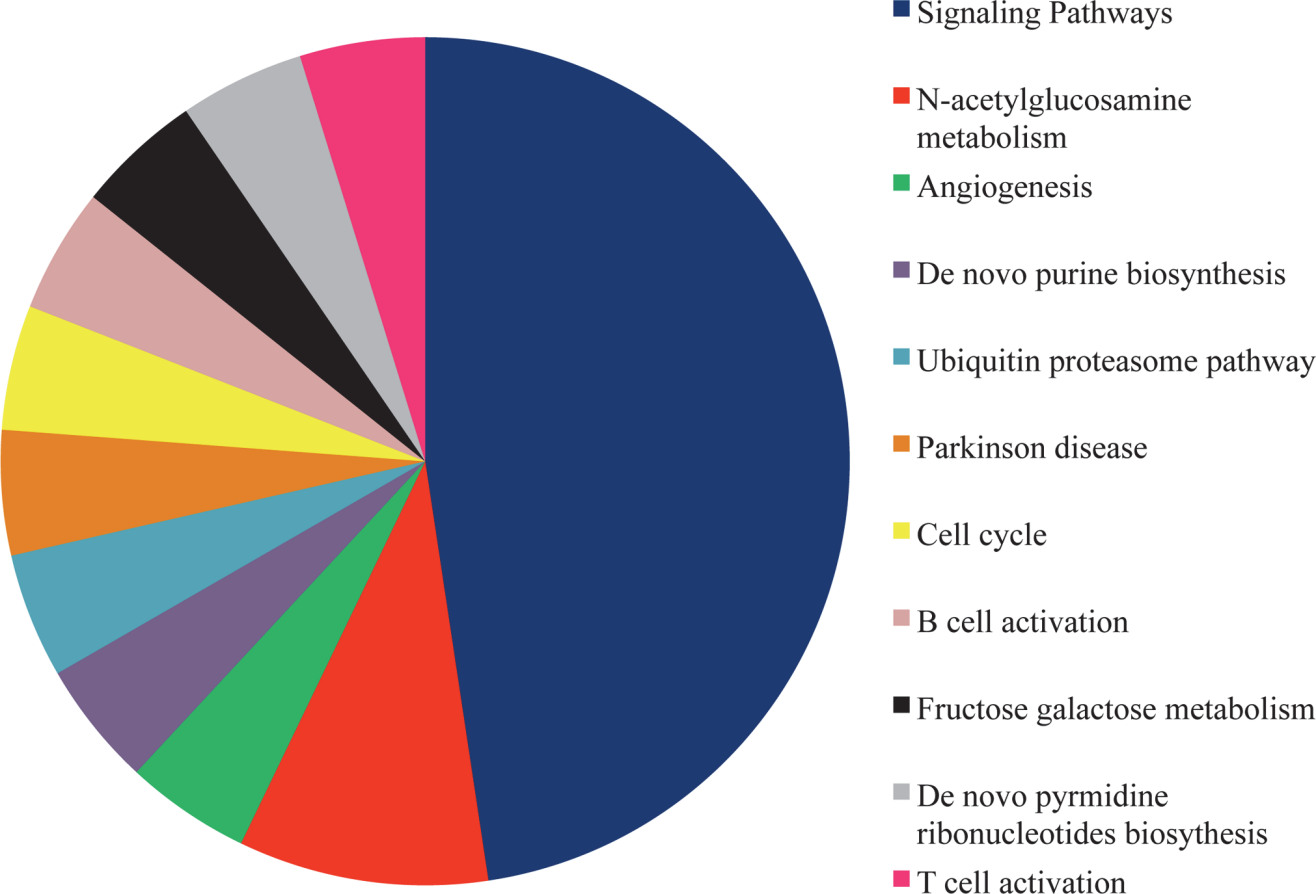
Pathway analysis performed using PANTHER. Panther gene ontology (GO) analysis for the 91 down-regulated target genes. Several metabolic pathways are affected, the majority of them is represented by signalling pathways, shaded in blue.

In parallel, ANalysisTHrough Evolutionary Relationship (PANTHER) showed that hsa-let-7g, hsa-let-7f, hsa-let7i and hsa-miR-125b up-regulation might determine changes in 29 metabolic pathways18 of which are signalling pathways (Fig. 5) mostly involving *RAF1*. Notably, *RAF1* is one of the 18 focus molecules identified by IPA in the network reported in Fig. 4A.

### *RAF1*, pERK1/2 and c-*Myc* expression in K562 FHC-silenced cells

**Fig 6:**
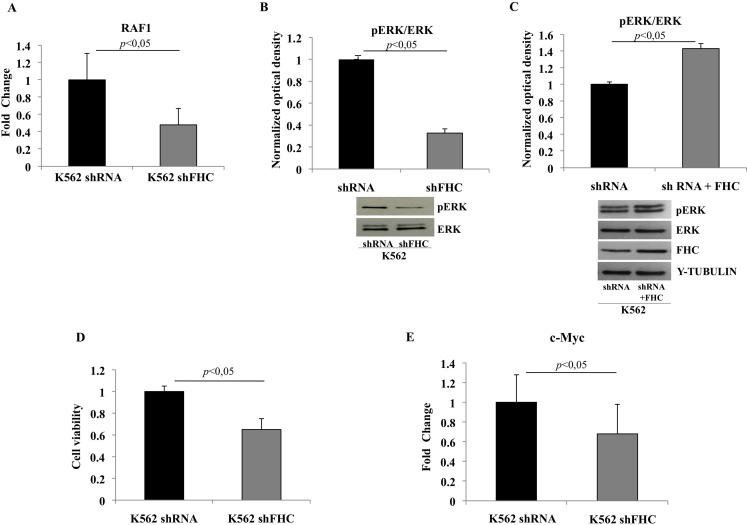
FHC silencing in K562 cells reduces proliferation rate via RAF1/MAPK pathway inhibition and is associated with *c-Myc*down-regulation. A) Real-time PCR of *RAF1* mRNA performed on K562 shRNA, K562 shFHC and K562^shFHC/pc3FHC^. Results are representative of two different experiments B) Western Blot analysis for pERK1/2 was performed on 50μg of total protein extract from K562shRNA and K562shFHC cells. Total ERK1/2 was used as loading control. Results are representative of three different experiments. C) Western Blot analysis for pERK1/2 and FHC was performed on 50μg of total protein extract from K562shRNA and K562shRNA+FHC. Total ERK1/2 and γ-Tubulin were used as loading controls. Results are representative of two different experiments. D) Equal number of starved silenced and un-silenced cells were plated into a 96-well plate, incubated for 72 h and analysed by MTT assay. Proliferation of FHC-silenced cells is reduced of about 35% compared to controls. Data are presented as mean ±standard deviation. E) Real-time PCR of c-*Myc* mRNA performed on K562 shRNA, K562 shFHC and K562^shFHC/pc3FHC^. Results are representative of two different experiments.

We noticed that, in the “Cell Death and Survival, Hematological System Development and Function, Hematopoiesis” network (Fig. 4A), the 18 focus molecules potentially modulated byhsa-let-7g-5p, hsa-let-7f-5p, hsa-let-7i-5p and hsa-miR-125b-5p, converge on a central hub represented by the ERK1/2 kinase. In particular, 6 of them directly impact on this kinase; *RAF-1*, *VEGFB*, *CD69* and *IRS2* are known activators, *PPP2CA* acts as inhibitor, while *SOCS1* might act either as inhibitor or activator depending on the cellular context. The potential involvement of ERK1/2 is further supported by the observation that the pathways identified by PANTHER analysis all depend on the activation of this molecule. Thus, we decided to investigate*RAF1* expression and ERK1/2 activation in the FHC-silenced cells by real-time PCR and western blot analysis, respectively. The experiments were performed in duplicate on RNAs and protein extracts from two independent sets of cells. Panel A of Fig. 6 shows that *RAF1* levels are significantly altered by FHC silencing, thus confirming the microarray data. Panel B shows that ERK1/2 phosphorylation is also severely impaired in the silenced cells compared to control. To further correlate ERK1/2 phosphorylation and FHC expression levels, we analysed pERK1/2 after FHC over-expression. Panel C of Fig. 6 shows that, in the control cells, an FHC over-expression of the order of about 37% is accompanied by an increased ERK1/2 phosphorylation. The role of ERK1/2 in the control of cell proliferation has been largely demonstrated [32]. Therefore, we analysed the proliferation rate of the silenced and un-silenced K562 cells by MTT assay. The experiments were performed in triplicate and the results, reported in Panel D of Fig. 6, indicate that the proliferation of FHC silenced cells is reduce of about 35% compared to the controls.

It has been reported that in the 3’ untranslated region of *c-Myc* mRNA there are multiple potential binding sites for Let-7 miRNAs family members. Moreover, the overexpression of Let-7 in cell cultures is accompanied by a decrease in *c-Myc* mRNA levels. Consequently, we determined by qRT-PCR the amounts of *c-Myc* mRNA in K562 cells silenced or not for FHC, finding that *c-Myc* mRNA was down-regulated to an extent of about 35% following FHC-silencing (Panel E of Fig. 6).

## Discussion

While the biochemical bases of ferritin function in iron uptake and deposition have been clearly established, and the respective roles of the two subunits determined, other aspects of its biological functions still remain to be clarified. Since the middle of last century, a robust body of data indicates that intracellular FHC is not only essential for iron metabolism but is also involved in critical metabolic pathways from the signalling cascades of CXCR4 [7] and G-CSFR [33] to Apo-B biogenesis [34].

We are interested in studying whether different intracellular amounts of FHC might affect gene expression profile of a given cell; proteome and transcriptome analysis has already revealed that the silencing of FHC is accompanied, in different cell types, by profound modifications in the steady-state amount of key proteins and transcripts [15, 27]. This phenomenon can be at least partially attributed to perturbations of the oxidative state of the cell induced by FHC-silencing, but the type and the amount of transcripts potentially regulated by FHCsuggest the existence of additional mechanisms that still require to be investigated. In this work, as first step toward the dissection of the molecular basis of FHC-modulated gene expression, we performed an integrated analysis of miRNA and mRNA expression patterns in K562 FHC-silenced cells.

We have utilised K562 cells in which the expression of FHC has been stably knocked-down by shRNA interference and whose transcriptome profile is already established [27]. By using a microRNA PCR Panel, we found that 4 out of 84 analysed miRNAs, namely hsa-let-7g-5p, hsa-let-7f-5p, hsa-let-7i-5p and hsa-miR-125b-5p, are consistently and significantly up-regulated in FHC-silenced K562 cells compared to control cells. The correlation among FHC amounts and the expression of the four miRNAs is further supported by transient silencing and reconstitution experiments.

The Let-7 human miRNA family is composed by 14 members widely considered as tumor suppressors.Let-7 miRNAs regulate, among others, the expression of the oncogenes *Ras*[35], *Myc*[36, 37] and *HMGA2*[38];accordingly, we found that, in FHC-silenced K562 cells, the up-regulation of Let7-g,-f and-i, is accompaniedby an important reduction of *Myc* expression.

Different members of the Let-7 family regulate highly overlapping set of genes, thus suggesting a redundant function. On the other hand, the regulation of their expression is elicited at multiple levels, and, in certain cancers, only specific members appear to be deregulated [39]. Therefore, an emerging question is whether different members of a miRNA family undergo a differential regulation within the same cell. Our data point in this direction, since FHC-silencing is accompanied, in K562 cells, by a selective up-regulation of three out of 9 Let-7 miRNAs analysed.

miR-125b, a member of the miR-125 family, is an intriguing molecule, acting either astumor suppressor or as an oncogene in different cancer types [40, 41]. Recently, miR-125b has been utilized as biomarker to distinguish cell lines derived from acute (HL60) and chronic (K562) myeloid leukemias and it has been proposed that in K562 it may act as a tumor promoting agent [42].

Our results demonstrate that *RAF1*, one of the target genes of miR-125b, is down-regulated in the FHC-silenced K562 cells. Moreover, we have shown, in these cells, a reduced activation of pERK1/2, that plays a central role in all the pathways in which the miRNA-regulated genes are involved. ERK1/2 MAP kinases regulate growth, survival and cell cycle progression in mammalian cells upon phosphorylation-induced activation [43]. Our data show that *FHC* knock-down may negatively regulate, through the modulation of miR-125b expression, the ERK activation thus suggesting that, in our experimental model, hsa-miR-125b may prevalently act as tumor suppressor molecule.Consistent with this hypothesis is also the significant reduction in proliferation rate of FHC-silenced cells. A correlation among FHC levels, has-miR-125b and ERK1/2 activation is further supported by the decreased miRNAs amount (data not shown) and the augmented phosphorylation of the MAPK in FHC over-expressing cells (Panel C of Fig. 6).

In this study, the profile of the four up-regulated miRNAs has been integrated with the transcriptome analysis by combining data obtained from the microRNA targets prediction software with a correlation-based approach. This test is based on the assumption that, since miRNAs tend to down-regulate the expression of their targets, the expression profiles of miRNAs are expected to be inversely related with those of their true target genes. This analysis led to the identification of 91 down-regulated genes, the majority of whom appear to be candidate targets of a single miRNA, while 15 are subjected to multiple miRNA regulation. IPA revealed that the highest scored pathways in which these genes are involved are: “Cell Death and Survival, Hematological System Development and Function, Hematopoiesis” and “DNA Replication, Recombination and Repair, Cell Cycle, Cancer”.The role of FHC in the processes of cell differentiation and neoplastic transformation has been investigated for a long time, starting from the observation that its intracellular amounts can significantly vary when comparing differentiated with undifferentiated cells, or transformed versus non transformed cells [44,5]. Both the central role of FHC in iron homeostasis and its ability in modulating different transduction pathways, are consistent with its increased expression during differentiation and neoplastic transformation. We believe that the identification of FHC-dependent miRNA/mRNA networks implies that different amounts of the ferritin subunit contribute, in K562 cells, to the remodelling of gene expression taking place during these cellular processes through the action of let-7g, let-7f, let-7i and miR-125b. This observation is further strengthened by the comparison of the miRNAs-regulated pathways, reported in this manuscript, with those highlighted in our previous work on FHC-silenced K562 undergoind differentiation [27]. It is interesting to note that, among the common pathways, “Cell Death and Survival” and “Hematological System Development and Function” rely on the ERK1/2 activation which is severely affected by FHC silencing.

In conclusion, the data presented in this study add a further level of complexity to the relationship among iron and miRNAs, demonstrating that the intracellular amounts of FHC subunit are able to regulate let-7g, let-7f, let-7i and miR-125b expression, as well as the repertoire of their down-stream genes. Even though an increasing body of evidence suggests that the redox state of the cell might significantly influence Let7 and 125-b levels [45, 46], we believe that the FHC interference on miRNA expression deserves further analysis.

## Supporting Information

S1 Table miRNAsexpression in FHC silenced K562 cells. List of 84 miRNAs.mi*RNAs* with an absolute Log fold-change greater than 2 are reported in bold.
(DOCX)

S2 Table Observed variations of gene expression after FHC silencing. The table shows the full cast of the FHC-dependent mRNA and their respective fold change of expression between K562 shFHC and K562 shRNA cells. The genes are ordered according to an increasing fold change.
(DOCX)

S3 Table miRNAs-mRNA significant correlation. The table reports 91 down-regulated target genes predicted by TargetScan analysis showing expression profiles significantly negatively correlated with that of the miRNAs (hsa-let-7g-5p, hsa-let-7f-5p, hsa-let-7i-5p and hsa-miR-125b-5p) significantly modulated after FHC silencing.
(DOCX)
