# Supplementary material for: H-Ferritin-Regulated MicroRNAs Modulate Gene Expression in K562 Cells
Source: PLoS One. 2015 Mar 27;10(3):e0122105. doi: 10.1371/journal.pone.0122105 (PMC4376865; doi:10.1371/journal.pone.0122105)
Supplement: S1 Table — List of 84 miRNAs.miRNAs with an absolute Log fold-change greater than 2 are reported in bold. (DOCX) [file pone.0122105.s001.docx]

**Table S1. miRNAs expression in FHC silenced K562 cells**

| **microRNA** | **LogFC**  **(shFHC vs shRNA)** |
| --- | --- |
| **hsa-miR-107** | **-3.33** |
| **hsa-miR-30d-5p** | **-2.55** |
| **hsa-miR-186-5p** | **-2.42** |
| hsa-miR-29c-3p | -1.69 |
| hsa-miR-9-5p | -1.50 |
| hsa-miR-31-5p | -1.34 |
| hsa-let-7c | -1.10 |
| hsa-miR-29a-3p | -1.04 |
| hsa-let-7b-5p | -0.73 |
| hsa-let-7e-5p | -0.68 |
| hsa-miR-130a-3p | -0.23 |
| hsa-miR-194-5p | -0.21 |
| hsa-miR-149-3p | -0.01 |
| hsa-miR-133a | 0.27 |
| hsa-miR-146a-5p | 0.31 |
| hsa-miR-22-3p | 0.43 |
| hsa-miR-145-5p | 0.50 |
| hsa-miR-148a-3p | 0.53 |
| hsa-miR-25-3p | 0.53 |
| hsa-miR-92b-3p | 0.55 |
| hsa-miR-26b-5p | 0.56 |
| hsa-miR-15b-5p | 0.58 |
| hsa-miR-93-5p | 0.59 |
| hsa-miR-29b-3p | 0.68 |
| hsa-miR-10b-5p | 0.73 |
| hsa-miR-7-5p | 0.75 |
| hsa-miR-103a-3p | 0.77 |
| hsa-miR-191-5p | 0.79 |
| hsa-miR-106b-5p | 0.80 |
| hsa-miR-26a-5p | 0.95 |
| hsa-miR-215 | 0.96 |
| hsa-miR-17-5p | 0.98 |
| hsa-miR-27a-3p | 1.04 |
| hsa-miR-223-3p | 1.07 |
| hsa-miR-202-3p | 1.07 |
| hsa-miR-23b-3p | 1.07 |
| hsa-miR-21-5p | 1.08 |
| hsa-miR-181a-5p | 1.17 |
| hsa-miR-423-5p | 1.19 |
| hsa-miR-1 | 1.21 |
| hsa-miR-100-5p | 1.21 |
| hsa-miR-143-3p | 1.21 |
| hsa-miR-155-5p | 1.21 |
| hsa-miR-200a-3p | 1.21 |
| hsa-miR-200b-3p | 1.21 |
| hsa-miR-205-5p | 1.21 |
| hsa-miR-20b-5p | 1.21 |
| hsa-miR-34a-5p | 1.21 |
| hsa-miR-192-5p | 1.23 |
| hsa-miR-210 | 1.26 |
| hsa-miR-132-3p | 1.31 |
| hsa-miR-195-5p | 1.35 |
| hsa-miR-19b-3p | 1.37 |
| hsa-miR-18a-5p | 1.37 |
| hsa-miR-214-3p | 1.37 |
| hsa-miR-221-3p | 1.39 |
| hsa-miR-19a-3p | 1.43 |
| hsa-miR-30c-5p | 1.45 |
| hsa-miR-200c-3p | 1.46 |
| hsa-miR-15a-5p | 1.48 |
| hsa-miR-20a-5p | 1.51 |
| hsa-miR-10a-5p | 1.54 |
| hsa-miR-182-5p | 1.54 |
| hsa-miR-106a-5p | 1.55 |
| hsa-miR-181b-5p | 1.59 |
| hsa-miR-23a-3p | 1.61 |
| hsa-miR-16-5p | 1.67 |
| hsa-miR-196a-5p | 1.72 |
| hsa-miR-27b-3p | 1.73 |
| hsa-miR-24-3p | 1.79 |
| hsa-miR-150-5p | 1.82 |
| hsa-miR-141-3p | 1.86 |
| **hsa-let-7a-5p** | **2.03** |
| **hsa-miR-222-3p** | **2.12** |
| **hsa-miR-206** | **3.45** |
| **hsa-miR-30b-5p** | **4.10** |
| **hsa-miR-101-3p** | **4.14** |
| **hsa-let-7i-5p** | **4.95** |
| **hsa-let-7d-5p** | **5.02** |
| **hsa-let-7f-5p** | **5.47** |
| **hsa-miR-125b-5p** | **5.82** |
| **hsa-miR-99a-5p** | **7.02** |
| **hsa-let-7g-5p** | **7.38** |
| **hsa-miR-126-3p** | **7.84** |
