# Supplementary material for: H-Ferritin-Regulated MicroRNAs Modulate Gene Expression in K562 Cells
Source: PLoS One. 2015 Mar 27;10(3):e0122105. doi: 10.1371/journal.pone.0122105 (PMC4376865; doi:10.1371/journal.pone.0122105)
Supplement: S2 Table — The table shows the full cast of the FHC-dependent mRNA and their respective fold change of expression between K562 shFHC and K562 shRNA cells. The genes are ordered according to an increasing fold change. (DOCX) [file pone.0122105.s002.docx]

**Table S2. Observed variations of gene expression after FHC silencing**

| **Gene** | **LogFC**  **(shFHC vs shRNA)** |
| --- | --- |
| HBZ | -8.49 |
| HBA2 | -7.07 |
| HIST2H2BF | -4.79 |
| GPR160 | -3.90 |
| TMEM14A | -3.73 |
| CDK5R1 | -3.18 |
| PPIG | -3.16 |
| MRPS5 | -3.15 |
| CETN2 | -3.14 |
| USP10 | -3.12 |
| CXXC1 | -3.11 |
| CEP44 | -3.09 |
| TP53TG3D | -3.08 |
| JARID2 | -3.05 |
| RHOT2 | -3.04 |
| LOC100506562 | -3.03 |
| EXOSC7 | -3.01 |
| BCKDK | -3.00 |
| COQ3 | -3.00 |
| CDC7 | -3.00 |
| MRPL46 | -2.95 |
| NAA10 | -2.89 |
| NUP153 | -2.89 |
| SPIN4 | -2.88 |
| HAUS7 | -2.85 |
| NOS3 | -2.85 |
| NAPRT1 | -2.85 |
| UAP1 | -2.82 |
| GFM1 | -2.80 |
| OCEL1 | -2.79 |
| NPRL3 | -2.74 |
| TATDN3 | -2.72 |
| ATOX1 | -2.68 |
| HIST1H3H | -2.67 |
| FAM98A | -2.66 |
| CENPK | -2.66 |
| UCHL3 | -2.63 |
| RPUSD3 | -2.63 |
| COL6A3 | -2.61 |
| PRMT5 | -2.61 |
| TBC1D7 | -2.56 |
| STUB1 | -2.56 |
| QDPR | -2.51 |
| FAM173A | -2.47 |
| MRPL20 | -2.37 |
| SPRR2F | -2.29 |
| NDUFB6 | -2.27 |
| RPP40 | -2.10 |
| TBCD | -2.08 |
| TXNRD1 | -2.07 |
| GSR | -2.06 |
| RPL35A | -2.04 |
| SNAR-A14 | -2.00 |
| PPAT | -1.95 |
| MELK | -1.87 |
| RPS20 | -1.85 |
| RPL38 | -1.85 |
| RPL27A | -1.84 |
| RN7SL1 | -1.83 |
| RPS16 | -1.81 |
| ANXA2P2 | -1.81 |
| GNB2L1 | -1.79 |
| GLCE | -1.78 |
| ZNF674 | -1.76 |
| CAV2 | -1.76 |
| EIF1AX | -1.72 |
| F2R | -1.71 |
| AMHR2 | -1.70 |
| RPL11 | -1.69 |
| LINC00857 | -1.60 |
| RPS5 | -1.58 |
| RPL27 | -1.58 |
| RRP7B | -1.58 |
| RPL13AP5 | -1.55 |
| UBC | -1.55 |
| ZNF644 | -1.55 |
| HBG1 | -1.55 |
| FEZ2 | -1.55 |
| LAIR1 | -1.55 |
| SLCO2B1 | -1.53 |
| FGFR3 | -1.52 |
| VWA5A | -1.51 |
| PPIAL4G | -1.50 |
| TDO2 | -1.49 |
| RPS10 | -1.49 |
| HBE1 | -1.49 |
| NPC2 | -1.44 |
| DNAJB6 | -1.44 |
| DGCR6L | -1.43 |
| HSP90AA1 | -1.42 |
| IFI27L1 | -1.41 |
| UQCRHL | -1.41 |
| HBG2 | -1.39 |
| GDF15 | -1.39 |
| RAB32 | -1.38 |
| NAV1 | -1.36 |
| GMFG | -1.35 |
| ATP5B | -1.30 |
| NEB | -1.27 |
| ECI2 | -1.25 |
| SLC50A1 | -1.25 |
| NUDT1 | -1.24 |
| ACYP2 | -1.19 |
| LOC729603 | -1.18 |
| MT1X | -1.18 |
| UQCC1 | -1.15 |
| PPM1E | -1.15 |
| SCAMP1 | -1.14 |
| MAOA | -1.13 |
| CCDC92 | -1.12 |
| CREBRF | -1.10 |
| ALOX12 | -1.07 |
| ACRBP | -1.05 |
| COX8A | -1.05 |
| DBI | -1.05 |
| ID2 | -1.04 |
| MGC57346 | -1.03 |
| RINL | -1.02 |
| HNRNPUL1 | 1.07 |
| ARHGAP22 | 1.07 |
| TEX11 | 1.08 |
| DDX17 | 1.09 |
| BTG3 | 1.13 |
| EIF3L | 1.13 |
| CGGBP1 | 1.14 |
| LOC728323 | 1.14 |
| PHKG1 | 1.22 |
| SERHL2 | 1.23 |
| RPS29 | 1.28 |
| EGFLAM | 1.34 |
| PRR3 | 1.42 |
| RPS9 | 1.45 |
| PNISR | 1.47 |
| PLTP | 1.48 |
| HBBP1 | 1.50 |
| PTGS1 | 1.50 |
| PIM2 | 1.52 |
| FAM127B | 1.53 |
| NEU1 | 1.57 |
| MT1G | 1.57 |
| CCPG1 | 1.59 |
| EXOSC1 | 1.66 |
| GLB1 | 1.67 |
| APOC1 | 1.67 |
| SKA2 | 1.68 |
| AP1S2 | 1.68 |
| PRCP | 1.70 |
| UBE2L3 | 1.72 |
| ATP2A2 | 1.73 |
| SDCBP | 1.74 |
| SH3GL3 | 1.75 |
| UBE2L6 | 1.76 |
| CCNB1IP1 | 1.85 |
| RPL23A | 1.87 |
| TRPC4AP | 1.98 |
| IL20RB | 2.01 |
| FAM129A | 2.02 |
| ID3 | 2.02 |
| BCL6 | 2.10 |
| CANX | 2.14 |
| ARHGDIB | 2.26 |
| JMJD8 | 2.29 |
| TPP1 | 2.32 |
| PCED1B | 2.32 |
| ANK1 | 2.34 |
| NFKBIA | 2.34 |
| RPL36 | 2.35 |
| IER3 | 2.36 |
| NT5C2 | 2.37 |
| LITAF | 2.40 |
| DCTD | 2.41 |
| SNX14 | 2.41 |
| PEA15 | 2.47 |
| NDRG1 | 2.48 |
| ZNF280A | 2.53 |
| LPIN1 | 2.54 |
| STX2 | 2.57 |
| CDC42EP4 | 2.60 |
| MTURN | 2.65 |
| H3F3B | 2.69 |
| CD36 | 2.74 |
| EHBP1 | 2.75 |
| TMEM173 | 2.78 |
| PIAS4 | 2.78 |
| DMPK | 2.78 |
| RPS23 | 2.81 |
| C14orf93 | 2.83 |
| CPNE3 | 2.84 |
| RENBP | 2.85 |
| SUGP1 | 2.85 |
| SERP1 | 2.86 |
| FLNB | 2.87 |
| PSMD5-AS1 | 2.89 |
| ACTN1 | 2.89 |
| HYOU1 | 2.91 |
| NBPF10 | 2.92 |
| NSMAF | 2.92 |
| MCM3AP | 2.95 |
| LRP10 | 2.95 |
| RBM5 | 2.96 |
| C15orf61 | 3.02 |
| DENND4B | 3.04 |
| HSBP1 | 3.06 |
| ELF1 | 3.07 |
| PRKAB1 | 3.07 |
| SLC52A2 | 3.08 |
| YIPF5 | 3.10 |
| AXIN1 | 3.11 |
| FZD5 | 3.19 |
| TCF3 | 3.19 |
| CLCF1 | 3.25 |
| EIF1 | 8.99 |
| JUND | 9.22 |
| TMEM258 | 9.33 |
| LRRC75A-AS1 | 9.35 |
| ZNF876P | 9.35 |
| ATP5O | 9.45 |
| KCNH6 | 9.67 |
| MT1H | 11.27 |
