# Supplementary material for: H-Ferritin-Regulated MicroRNAs Modulate Gene Expression in K562 Cells
Source: PLoS One. 2015 Mar 27;10(3):e0122105. doi: 10.1371/journal.pone.0122105 (PMC4376865; doi:10.1371/journal.pone.0122105)
Supplement: S3 Table — The table reports 91 down-regulated target genes predicted by TargetScan analysis showing expression profiles significantly negatively correlated with that of the miRNAs (hsa-let-7g-5p, hsa-let-7f-5p, hsa-let-7i-5p and hsa-miR-125b-5p) significantly modulated after FHC silencing. (DOCX) [file pone.0122105.s003.docx]

**Table S3. miRNAs-mRNA significant correlation**

| **Gene** | **miRNA** | **Correlation** | **p-value** |
| --- | --- | --- | --- |
| SEC14L1 | hsa-let-7f | -1.000 | 0.003 |
| GANAB | hsa-let-7f | -1.000 | 0.003 |
| SEMA4F | hsa-let-7f | -1.000 | 0.003 |
| HABP4 | hsa-let-7f | -0.943 | 0.017 |
| DLGAP4 | hsa-let-7f | -0.943 | 0.017 |
| SOCS1 | hsa-let-7f | -0.943 | 0.017 |
| USP32 | hsa-let-7f | -0.943 | 0.017 |
| FAM118A | hsa-let-7f | -0.943 | 0.017 |
| GIPC1 | hsa-let-7f | -0.943 | 0.017 |
| ZNF10 | hsa-let-7f | -0.886 | 0.033 |
| RDX | hsa-let-7f | -0.886 | 0.033 |
| SFMBT1 | hsa-let-7f | -0.886 | 0.033 |
| IRS2 | hsa-let-7f | -0.886 | 0.033 |
| PARP8 | hsa-let-7f | -0.886 | 0.033 |
| LIPT2 | hsa-let-7f | -0.886 | 0.033 |
| PPARGC1B | hsa-let-7f | -0.886 | 0.033 |
| MAN2A2 | hsa-let-7f | -0.886 | 0.033 |
| WDR73 | hsa-let-7f | -0.886 | 0.033 |
| HBEGF | hsa-let-7f | -0.886 | 0.033 |
| GJC1 | hsa-let-7f | -0.812 | 0.050 |
| IRS2 | hsa-let-7g | -1.000 | 0.003 |
| PARP8 | hsa-let-7g | -1.000 | 0.003 |
| MAN2A2 | hsa-let-7g | -1.000 | 0.003 |
| HBEGF | hsa-let-7g | -1.000 | 0.003 |
| HABP4 | hsa-let-7g | -0.943 | 0.017 |
| HAND2 | hsa-let-7g | -0.943 | 0.017 |
| CMTM6 | hsa-let-7g | -0.943 | 0.017 |
| DLGAP4 | hsa-let-7g | -0.943 | 0.017 |
| FAM118A | hsa-let-7g | -0.943 | 0.017 |
| HIST2H2BF | hsa-let-7g | -0.943 | 0.017 |
| GIPC1 | hsa-let-7g | -0.943 | 0.017 |
| GALNT1 | hsa-let-7g | -0.943 | 0.017 |
| CTPS1 | hsa-let-7g | -0.886 | 0.033 |
| TRIM5 | hsa-let-7g | -0.886 | 0.033 |
| SEC14L1 | hsa-let-7g | -0.886 | 0.033 |
| FAM214B | hsa-let-7g | -0.886 | 0.033 |
| EPB41L4A | hsa-let-7g | -0.886 | 0.033 |
| AMT | hsa-let-7g | -0.886 | 0.033 |
| GANAB | hsa-let-7g | -0.886 | 0.033 |
| OSBPL3 | hsa-let-7g | -0.886 | 0.033 |
| USP24 | hsa-let-7g | -0.886 | 0.033 |
| SEMA4F | hsa-let-7g | -0.886 | 0.033 |
| RMI2 | hsa-let-7g | -0.886 | 0.033 |
| GALE | hsa-let-7g | -0.886 | 0.033 |
| SLC25A12 | hsa-let-7g | -0.886 | 0.033 |
| DDX26B | hsa-let-7i | -0.943 | 0.017 |
| ATP8B4 | hsa-let-7i | -0.943 | 0.017 |
| TGFBR3 | hsa-let-7i | -0.943 | 0.017 |
| NXT2 | hsa-let-7i | -0.943 | 0.017 |
| UTRN | hsa-let-7i | -0.943 | 0.017 |
| SLC35D2 | hsa-let-7i | -0.928 | 0.008 |
| DLGAP4 | hsa-let-7i | -0.886 | 0.033 |
| PAG1 | hsa-let-7i | -0.886 | 0.033 |
| ELF4 | hsa-let-7i | -0.886 | 0.033 |
| GXYLT1 | hsa-let-7i | -0.886 | 0.033 |
| FAM118A | hsa-let-7i | -0.886 | 0.033 |
| NAGA | hsa-let-7i | -0.886 | 0.033 |
| GIPC1 | hsa-let-7i | -0.886 | 0.033 |
| MEGF9 | hsa-miR-125b | -1.000 | 0.003 |
| RAB43 | hsa-miR-125b | -1.000 | 0.003 |
| TDG | hsa-miR-125b | -0.943 | 0.017 |
| SPATA31C1 | hsa-miR-125b | -0.943 | 0.017 |
| CTPS1 | hsa-miR-125b | -0.943 | 0.017 |
| P2RX4 | hsa-miR-125b | -0.943 | 0.017 |
| CD69 | hsa-miR-125b | -0.943 | 0.017 |
| ST6GAL1 | hsa-miR-125b | -0.943 | 0.017 |
| FAM53C | hsa-miR-125b | -0.943 | 0.017 |
| PPAT | hsa-miR-125b | -0.943 | 0.017 |
| CCDC126 | hsa-miR-125b | -0.943 | 0.017 |
| UBA52 | hsa-miR-125b | -0.943 | 0.017 |
| ARL6IP4 | hsa-miR-125b | -0.943 | 0.017 |
| XRCC3 | hsa-miR-125b | -0.943 | 0.017 |
| P2RX6 | hsa-miR-125b | -0.943 | 0.017 |
| GNPDA1 | hsa-miR-125b | -0.943 | 0.017 |
| NEO1 | hsa-miR-125b | -0.943 | 0.017 |
| ICAM2 | hsa-miR-125b | -0.943 | 0.017 |
| PPP2CA | hsa-miR-125b | -0.943 | 0.017 |
| PSTPIP2 | hsa-miR-125b | -0.943 | 0.017 |
| ABHD6 | hsa-miR-125b | -0.943 | 0.017 |
| PHF23 | hsa-miR-125b | -0.943 | 0.017 |
| PSMD7 | hsa-miR-125b | -0.943 | 0.017 |
| SNX25 | hsa-miR-125b | -0.943 | 0.017 |
| ATP5G2 | hsa-miR-125b | -0.943 | 0.017 |
| VEGFB | hsa-miR-125b | -0.943 | 0.017 |
| ADRBK1 | hsa-miR-125b | -0.943 | 0.017 |
| RAF1 | hsa-miR-125b | -0.943 | 0.017 |
| SPRTN | hsa-miR-125b | -0.943 | 0.017 |
| ARHGEF2 | hsa-miR-125b | -0.943 | 0.017 |
| AZI2 | hsa-miR-125b | -0.943 | 0.017 |
| SEMA3E | hsa-miR-125b | -0.943 | 0.017 |
| SNX22 | hsa-miR-125b | -0.943 | 0.017 |
| GALE | hsa-miR-125b | -0.943 | 0.017 |
| SLC46A3 | hsa-miR-125b | -0.943 | 0.017 |
| GPR160 | hsa-miR-125b | -0.928 | 0.008 |
| WARS | hsa-miR-125b | -0.886 | 0.033 |
| ASXL2 | hsa-miR-125b | -0.886 | 0.033 |
| ARRB1 | hsa-miR-125b | -0.886 | 0.033 |
| VSTM4 | hsa-miR-125b | -0.886 | 0.033 |
| BMF | hsa-miR-125b | -0.886 | 0.033 |
| ZNF263 | hsa-miR-125b | -0.886 | 0.033 |
| HIST2H2BF | hsa-miR-125b | -0.886 | 0.033 |
| SNX24 | hsa-miR-125b | -0.886 | 0.033 |
| TMEM50A | hsa-miR-125b | -0.886 | 0.033 |
| SZRD1 | hsa-miR-125b | -0.886 | 0.033 |
| CRCP | hsa-miR-125b | -0.886 | 0.033 |
| QSOX2 | hsa-miR-125b | -0.886 | 0.033 |
| GPR153 | hsa-miR-125b | -0.886 | 0.033 |
| TMEM87A | hsa-miR-125b | -0.812 | 0.050 |
